# CUREMA project: a further step towards malaria elimination among hard-to-reach and mobile populations

**DOI:** 10.1186/s12936-024-05040-8

**Published:** 2024-09-10

**Authors:** Alice Sanna, Yann Lambert, Irene Jimeno Maroto, Muriel Suzanne Galindo, Lorraine Plessis, Teddy Bardon, Carlotta Carboni, Jane Bordalo, Helene Hiwat, Hedley Cairo, Lise Musset, Yassamine Lazrek, Stéphane Pelleau, Michael White, Martha Suárez Mutis, Stephen Vreden, Maylis Douine

**Affiliations:** 1 French West Indies-French Guiana Center for Clinical Investigation (CIC Inserm 1424), Department of Research, Innovation, and Public Health, Cayenne Hospital, Cayenne, French Guiana France; 2 Associação Desenvolvimento, Prevenção, Acompanhamento e Cooperação de Fronteiras (DPAC), Oiapoque, Brazil; 3 National Malaria Programme, Ministry of Health, Paramaribo, Suriname; 4 Laboratoire de Parasitologie, Institut Pasteur de la Guyane, Centre National de Référence du Paludisme, Cayenne, French Guiana France; 5 Infectious Disease Epidemiology and Analytics, Institut Pasteur, Université Paris Cité, Paris, France; 6 Laboratory of Parasitic Diseases, Graduate Program in Tropical Medicine, Instituto Oswaldo Cruz, Fiocruz, Rio de Janeiro, Brazil; 7 Foundation for the Advancement of Scientific Research in Suriname (SWOS), Paramaribo, Suriname

**Keywords:** Malaria, Elimination, Research protocol, Public health intervention, Cross-border, Mobility, Hard-to-reach population, *Plasmodiumvivax*, Radical cure

## Abstract

**Background:**

In most countries engaged on the last mile towards malaria elimination, residual transmission mainly persists among vulnerable populations represented by isolated and mobile (often cross-border) communities. These populations are sometimes involved in informal or even illegal activities. In regions with *Plasmodium vivax* transmission, the specific biology of this parasite poses additional difficulties related to the need for a radical treatment against hypnozoites to prevent relapses. Among hard-to-reach communities, case management, a pillar of elimination strategy, is deficient: acute malaria attacks often occur in remote areas, where there is limited access to care, and drugs acquired outside formal healthcare are often inadequately used for treatment, which typically does not include radical treatment against *P. vivax*. For these reasons, *P. vivax* circulation among these communities represents one of the main challenges for malaria elimination in many non-African countries. The objective of this article is to describe the protocol of the CUREMA study, which aims to meet the challenge of targeting malaria in hard-to-reach populations with a focus on *P. vivax*.

**Results:**

CUREMA is a multi-centre, international public health intervention research project. The study population is represented by persons involved in artisanal and small-scale gold mining who are active and mobile in the Guiana Shield, deep inside the Amazon Forest. The CUREMA project includes a complex intervention composed of a package of actions: (1) health education activities; (2) targeted administration of treatment against *P. vivax* after screening against G6PD deficiency to asymptomatic persons considered at risk of silently carrying the parasite; (3) distribution of a self-testing and self-treatment kit (malakit) associated with user training for self-management of malaria symptoms occurring while in extreme isolation. These actions are offered by community health workers at settlements and neighbourhoods (often cross-border) that represent transit and logistic bases of gold miners. The study relies on hybrid design, aiming to evaluate both the effectiveness of the intervention on malaria transmission with a pre/post quasi-experimental design, and its implementation with a mixed methods approach.

**Conclusions:**

The purpose of this study is to experiment an intervention that addresses both *Plasmodium falciparum* and *P. vivax* malaria elimination in a mobile and isolated population and to produce results that can be transferred to many contexts facing the same challenges around the world.

## Background

In 2022, the worldwide number of malaria cases was estimated at 249 million, causing approximately 608000 deaths [1]. While global scaling-up of malaria control interventions led to apparent decrease between 2000 and 2015, the main indicators of morbidity and mortality remained broadly stable between 2015 and 2020, and even increased after the Covid-19 pandemic. This suggests that the limits of current strategies have been reached and that new methods need to be developed, both in terms of technology and of operational approaches, to achieve the target 90% reduction of malaria morbidity and mortality by 2030, as set by WHO Global malaria technical Strategy [2].

*Plasmodium vivax* is the second malaria parasite species by order of incidence on a global scale, with an estimated 6.9 million cases per year in the World [1]. It is currently responsible for two thirds of malaria cases in the Americas, with up to 80% relapses [3]. The only means of prevention of relapses is a radical pharmacological treatment by a cure of 8-aminoquinoline drugs (primaquine for 7 or 14 days or, recently, a single-dose of tafenoquine) [4–6]. Testing for G6PD deficiency (a red blood cell hereditary condition) is recommended before administering this radical treatment, in order to avoid serious adverse reactions such as haemolysis [5]. No direct diagnostic test is available to detect *P. vivax* hypnozoites carriage, meaning that it is impossible to identify a latent infection when blood-stage parasites are no longer detectable [7]. For all these reasons, territories endemic for *P. vivax* usually struggle with access to radical treatment for all affected individuals [8].

In territories progressing toward malaria elimination, a typical transition in epidemiology is often observed: spatial heterogeneity becomes more marked, the proportion of cases caused by *P. vivax* more important, and adult males become the most affected sub-population [9–11]. Spatial strata characterized by more intense transmission combine favourable environmental characteristics (including recent anthropization of natural environments), geographic isolation, human mobility (particularly in cross border contexts), and often occupational and socio-economic and/or political vulnerability [9, 10, 12–14]. Indeed, among mobile and isolated populations, case management, is often deficient: attacks often occur in remote areas with no access to care and treatment is often inadequate with smuggled drugs, usually not including *P. vivax* anti-hypnozoite treatment [15, 16]. These individuals might be asymptomatic carriers of *Plasmodium,* due to acquired partial immunity [17–19] and thus contribute to sustaining transmission in remote rural or forested areas, but also in urban and peri-urban settings through continuous spillover [20–24]. In this light, populations living in remote areas represent hotspots of *P. vivax* transmission, and *Plasmodium* clearance among mobile and isolated groups is the ultimate challenge for malaria elimination in many low and medium transmission settings [9]. *Plasmodium vivax* patients should be rationally treated with radical therapy; irrational drug use, such as with smuggled drugs is discouraged.

In the Amazon region, persons involved in artisanal and small-scale gold mining (ASGM) are a typical hard-to-reach population [25]. They live and work for weeks or months deep in the rainforest, where the density of malaria vectors (*Anopheles* spp.) is high [20, 25, 26]*.* As they are often involved in informal or illegal mining, national health systems may be unable to implement specific interventions to reach them, due to unfavourable regulations or an unsupportive political environment. These miners regularly move to other areas in search of more productive sites, or for logistical or personal reasons. Gold mining areas are often characterized by high malaria endemicity, and miners can fuel malaria reintroduction in low burden areas [25, 27–30]. Their mobility is often cross-border or transnational in the Amazon region, making it a complex challenge to individual as well as public health management [25, 28, 31].

French Guiana (FG) is the only territory in the European Union where indigenous transmission of malaria is currently ongoing. It is located within the Guiana Shield and shares land borders with Brazil (Amapá State) and Suriname [32, 33]. FG, Amapá State and Suriname share a common decreasing malaria incidence, a predominance of *P. vivax* and transmission mainly concentrated in gold mining areas and in some cases in remote indigenous communities [32, 34, 35]. In this region, persons involved in ASGM (*garimpeiros)* are mainly of Brazilian origin, and are highly mobile across the Guiana Shield [25, 28, 31, 36, 37]. The World Health Organization (WHO) included Suriname and FG among the territories that could defeat malaria by 2025 (E-2025 initiative) [1].

A first public health intervention research project, Malakit, was implemented from 2018 to 2020 at the borders between FG and Brazil and Suriname to address access to malaria diagnostic testing and good quality treatment for persons working in remote and illegal mines in FG [20, 36, 38, 39]. The project’s intervention consisted in making available a kit, including malaria rapid diagnostic tests (RDTs) and an artemisinin-based combination therapy (ACT), as well as a training on how to correctly self-test and self-treat delivered at the *garimpeiros*’ cross-border staging areas by community health workers (CHWs). This study, evaluating an innovative intervention, has constituted an urgent and pragmatic response to the risk of emergence of resistant *P. falciparum* linked to inappropriate use of smuggled ACT doses among the target population [15, 40, 41]. The project’s strategy showed to be successful: the proportion of *garimpeiros* reporting proper treatment with an ACT after a positive RDT significantly increased (OR = 1.8 95% CI [1.1–3.0]) [39]. Mathematical modelling estimates that the Malakit project helped prevent 43% of the cases imported from FG to Brazil and Suriname [39, 42]. However, the Malakit intervention does not offer a solution to prevent *P. vivax* relapses: while the overall malaria prevalence and incidence decreased, the proportion of *P. vivax* infection among the target population increased after the intervention (from 42 to 85% among persons recruited at the FG-Suriname border, and from 85.7% to 100% at the FG-Brazil border) [39].

Recently, several tools have joined the arsenal against *P. vivax* malaria*.* Tafenoquine has been approved for *P. vivax* radical cure by health authorities from an increasing number of endemic countries [43]. This drug has stricter contraindications because of its long half-life and a higher haemolytic risk in case of G6PD deficiency [44, 45], but its use at a single-dose presents an important advantage compared to primaquine, which is subject to sub-optimal adherence even with a short 7 day regimen [46, 47]. A recent point-of-care device for quantitative evaluation of G6PD activity [48–50] has performed very well in identifying severe and intermediate G6PD deficiency compared to the gold standard. It has been successfully tested in the field in Asian countries [51–53] and in Brazil [54–56], and allows for field implementation of tafenoquine treatment. The roll out of this innovative technology in the routine of health care services is currently being planned and implemented in the territories of the Guiana Shield.

Considering the evolution of the malaria epidemiology with a predominance of *P. vivax* among *garimpeiros*, the importance to tailor specific strategies to reach this population, and the availability of new tools for *P. vivax* radical cure, a new interventional project called CUREMA (*Radical CURE for MAlaria among highly mobile and hard-to-reach populations in the Guiana Shield*) has been designed. The aim of this project is to evaluate an intervention targeting malaria elimination (*P. falciparum* and *P. vivax*) among the persons working in ASGM in the Region.

This article presents the protocol of the CUREMA project.

## Methods

The CUREMA project is a mixed-methods interventional, multicentric, international study.

It aims at evaluating a new public health intervention targeting malaria among hard-to-reach and mobile populations [57]. The main objectives of the project are:

- To evaluate the impact of the intervention on malaria transmission among persons involved in ASGM in the Guiana Shield.
- To evaluate the implementation of the intervention and to identify obstacles and levers to inform on transferability and scaling-up.

### Intervention’s target population

The target population of the intervention is represented by people actively involved in ASGM in the Region. Active participation in gold mining is defined as having worked in a gold mine in the last 12 months, or planning to enter a gold mine in the next month. As described in previous publications [20, 36, 38] the population is predominantly male (around three quarters), adult, and is involved in a variety of activities: the various aspects of metal extraction and site management, support services, such as cooking, sales (through small grocery stores or as mobile vendors), transport of people or goods (by river or land, with portage or ATVs), mechanics, wood removal for site structures, and sex work.

Criteria for participation in the study are summarized in Table 1.

**Table 1:** Inclusion, eligibility and exclusion criteria for the intervention

Inclusion criteria		Eligibility criteria	Exclusion criteria
Be 18 years of age or older Agree to participate in the study Have a current involvement in gold mining activities (having been to the *garimpo * in the last year or planning to enter the *garimpo* in the following month), regardless of country No symptoms of malaria at the time of the inclusion visit Weigh over 35 kg	Radical cure service	-Wish to receive this service -Epidemiological criteria in favour of a current asymptomatic carriage of *P. vivax* (blood stage or liver stage). At least one of the following conditions: o have a history of clinical malaria during the past 12 months o OR having a life-long malaria history AND have stayed for at least 1 week during the last 12 months in an area with extensive *P. vivax* transmission	-Refuse to participate in an active follow-up during the 14 days following the start of treatment -Current pregnancy (declared or rapid urine test positive) or breastfeeding -Haemoglobinemia below 9 g/dL -G6PD activity below 70% (*for simplicity G6PD activity of 6 IU/dL or below based on STANDARD*^*™*^ *G6PD analyzer output*) -Have received a unique dose of tafenoquine within the last 3 months or a full course of primaquine within the last month -Hypersensitivity or known contraindication to chloroquine, primaquine or tafenoquine -History of severe mental health disorder -Having a positive malaria rapid diagnostic the day of the inclusion or currently receiving an anti-malarial treatment
	Malakit service	-Wish to receive this service -Plan to enter a *garimpo* located in French Guiana the following month	-Inability to self-test (perform and interpret an RDT) during training -Inability to understand and explain correctly what to do in case of malaria symptoms (test and ACT posology)

### Study settings

**Fig. 1:**
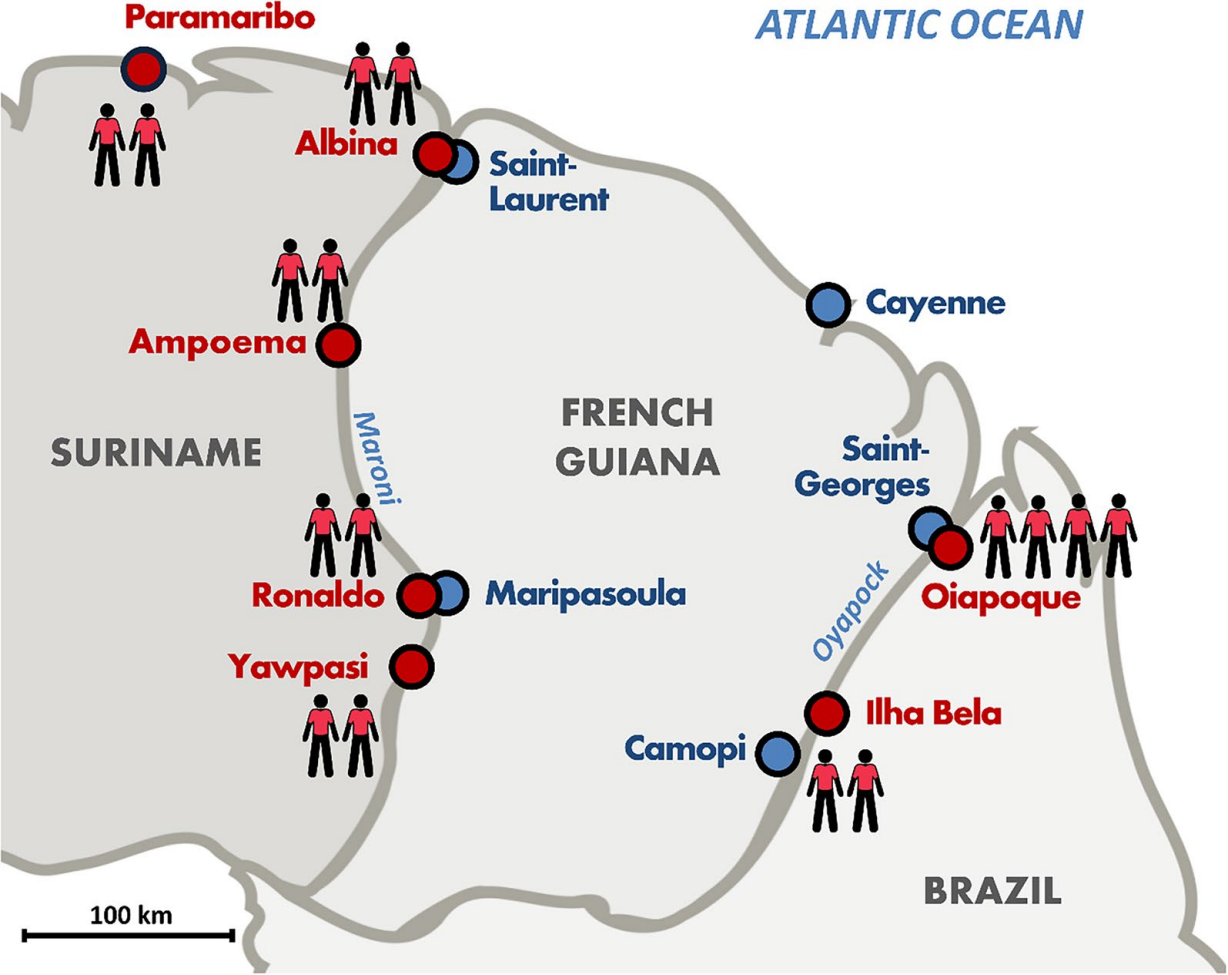
Map illustrating the study locations (red dots) and the respective project’s field teams

The study is carried out in Suriname and Brazil (Amapá State). The study’s facilities and inclusion sites are mainly located at cross-border points (towns or small informal settlements located on the riverbanks) along the two river borders of FG, considered crossing points and logistics hubs for the target population, where can be found shops, bars and accommodation facilities mainly receiving *garimpeiros*. These are “neutral” places where the public is easy to meet and not in a clandestine situation. They are illustrated in Fig. 1.

### CUREMA intervention and its implementation strategy

#### Intervention

The CUREMA intervention is a package of actions including three components: two different services offered to participants, with a common core component of health education.

The health education activities focus on malaria: its causes, means of prevention, the main differences between *P. falciparum* and *P. vivax*, and the importance of a complete anti-malarial treatment. It is provided to participants as part of the inclusion process in the study, and to the community during out-reach activities.

**Fig. 2:**
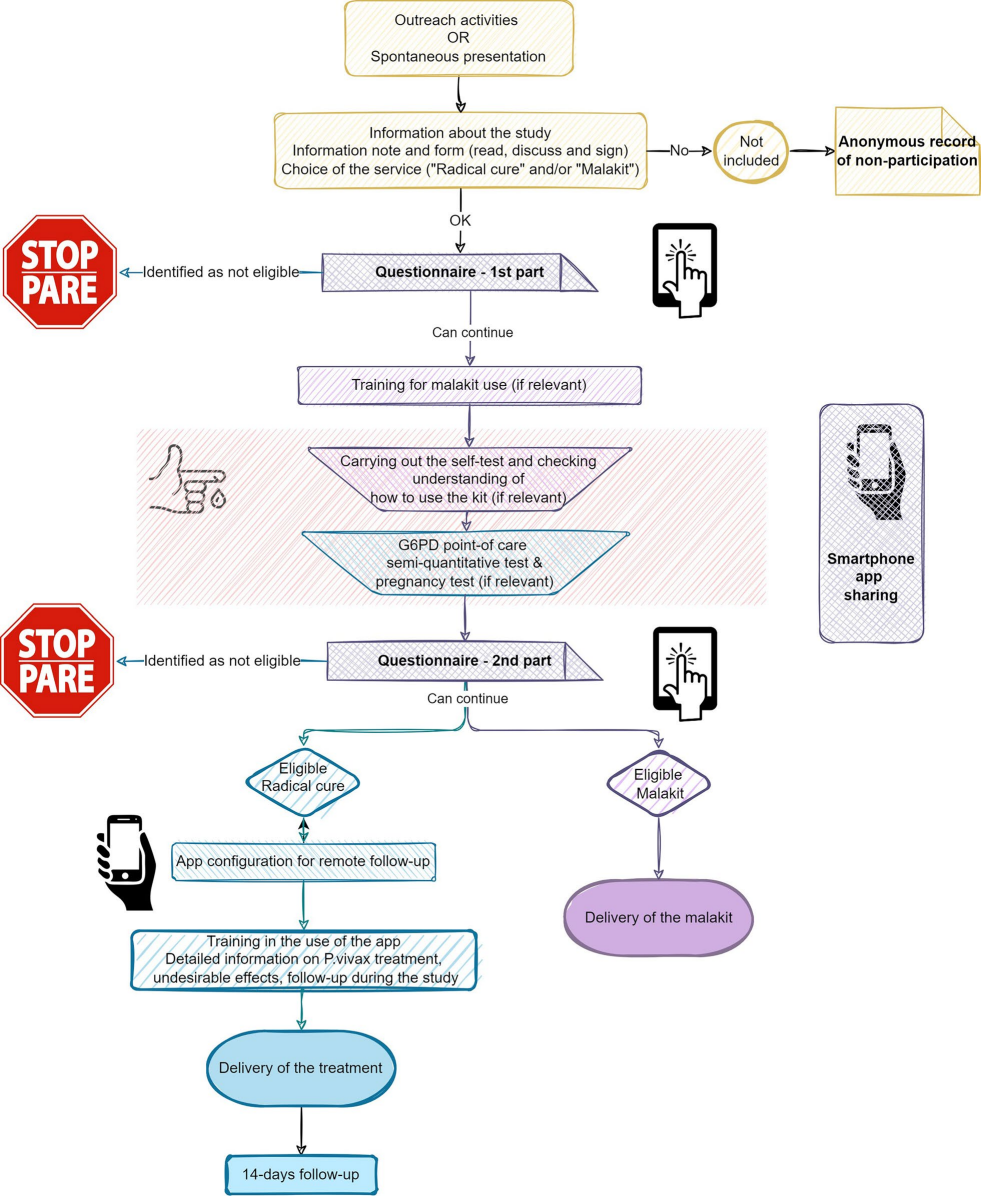
Inclusion process for the CUREMA intervention

Each participant, after collection of written and informed consent, is able to choose whether to participate in one or both services: the “radical cure” and the “malakit”. During the inclusion process (Fig. 2), the participants answer a short questionnaire designed (1) to collect socio-demographic and occupational data, and (2) to assess eligibility criteria to the service(s) selected by the participants.

The *“radical cure”* represented by the treatment of asymptomatic individuals considered at risk of carrying *P. vivax* hypnozoites. The objective of this service is to prevent relapses and thus to reduce further transmission of this parasite.

Individuals considered at risk of carrying *P. vivax* are identified through questions from the inclusion questionnaire, regarding their recent exposure to malaria. Contra-indications to radical cure are also documented within the questionnaire (breastfeeding, history of allergy or other side effect to 8-aminoquinoline or chloroquine, severe mental health disorders history) and point-of-care tests: quantitative assessment of G6PD activity level performed with capillary blood through STANDARD G6PD tests from SD Biosensor performed by CHWs, and urine pregnancy test for women of childbearing age.

**Fig. 3:**
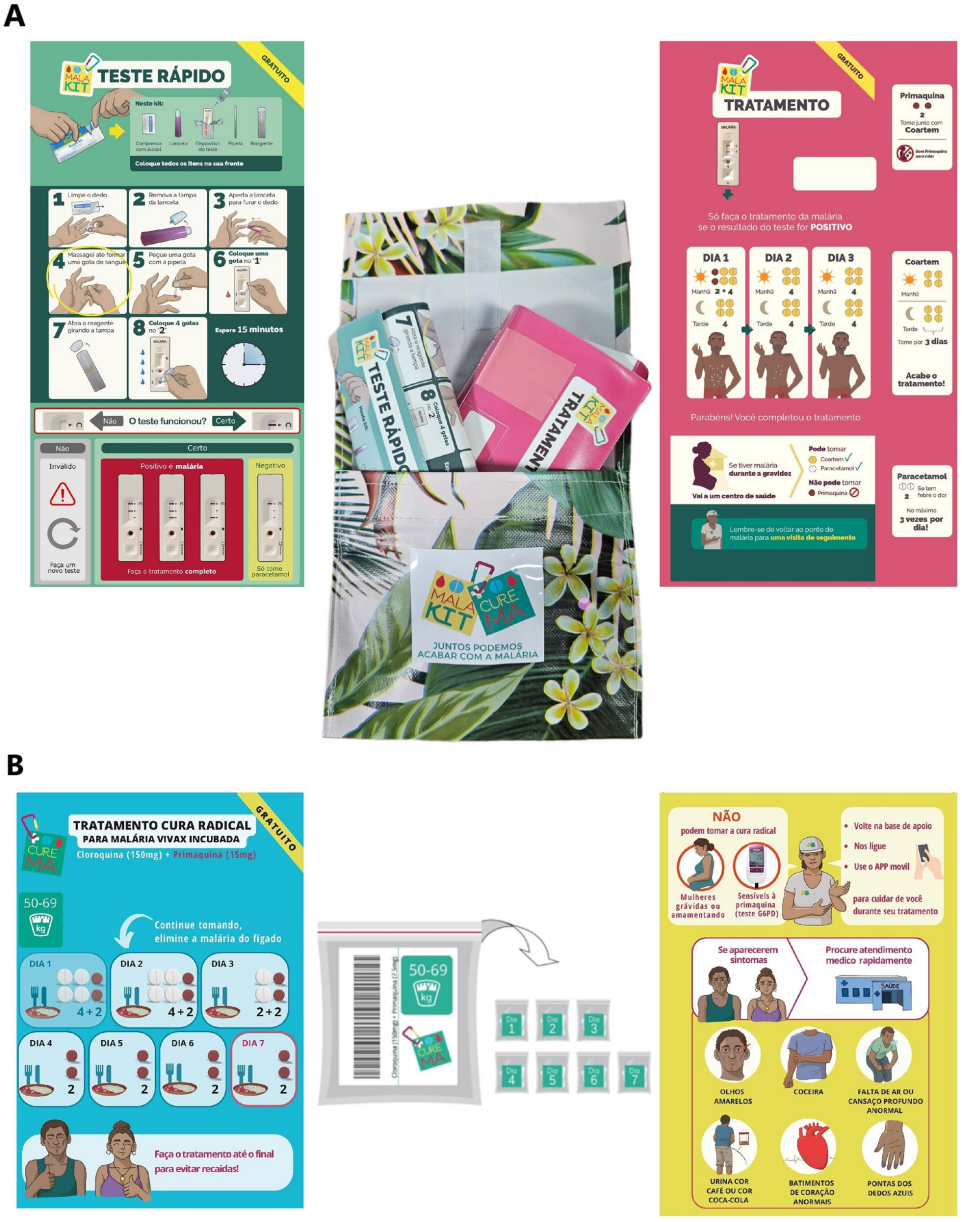
Kits for participants to the CUREMA intervention: **A** self-test and self treatment kit, called malakit, consisting in a test pocket (green) and a treatment kit (pink), both illustrated in order to guide kit use by illiterate participants; **B** radical cure kit, consisting of a treatment pocket (with numbered Ziploc with daily treatment doses) and illustrated flyers informing on posology, contra-indication and potential side effects

Eligible participants receive a three-day course of chloroquine associated to an 8-aminoquinoline drug (a 7 day course of primaquine 0.5 mg/kg/day adjusted by weight categories, or a unique dose of 300 mg of tafenoquine). The treatment is started immediately, and the first dose uptake is directly observed. During the inclusion process participants receive oral and written instructions on how to take the tablets, potential side effects and what to do in case of an adverse event (AE), including the potential need to seek urgent care (Fig. 3).

Adherence and safety data are collected by a 14 day follow-up. Follow-up visits (planned at 2, 5 and 14 days after the start of the treatment) are ensured by several tools tailored to the context and to the usual short-term mobility of the target population: an in-person or phone follow-up by CHWs, or self-reporting via a smartphone application. In both cases the follow-up consists in a short questionnaire exploring the main symptoms of significant AEs (haemolysis, allergy, cardiac rhythm modifications). In order to detect any further serious adverse events, participants are also asked about their perceived general state of health, and whether they have had to seek medical attention since starting treatment. In case of positive answer to either one of these questions, the participant is invited to stop the treatment and to seek care at the nearest health facility; an interview is also performed by one of the physician investigators of the study and, if deemed necessary, further clinical and biological explorations are proposed to assess (1) the severity of the AE and (2) the causal link with the medications delivered in the context of the study. Serious adverse events (e.g. severe haemolysis) are collected and reported immediately to the relevant authorities, the sponsor's pharmacovigilance team and the Data and Safety Monitoring Board of the study.

The 8-aminoquinoline initially implemented at inclusion sites is primaquine; tafenoquine will be gradually introduced in the inclusion process as soon as the field procedures of inclusion and follow-up are robust and the drug available (donation by GSK).

*The malakit* represented by the delivery, after appropriate training, of a self-testing and self-treatment kit. The objective of this service is to provide access to quality diagnosis and treatment for episodes of symptoms compatible with malaria that occur in situations of extreme remoteness from health services. The kit is composed of two illustrated plastic pouches. The diagnostic pouch contains three malaria rapid diagnostic tests Bioline malaria rapid tests by Abbott, chosen because they are prequalified by the WHO, have individual packaging and are capable of detecting the malaria species circulating in the region; the models used depend on the purchasing possibilities of the countries concerned according to the local regulations. The treatment pouch contains a blister of paracetamol, a full course of artemether-lumefantrine (20 mg/120 mg) and a single low dose of primaquine (15 mg) to target *P. falciparum* gametocytes and prevent onwards transmission (Fig. 3) [39]. Participants receive training about malaria symptoms, how to correctly perform rapid tests and how to follow the treatment. Knowledge assessment is carried out after the training, and participants have to perform and interpret a self-test correctly in order to be eligible to receive the kit.

#### Implementation strategy

The aim of this study is to evaluate both the intervention and its implementation under the conditions relevant to the target population and context. It is, therefore, essential to describe in systematic manner the main features of the chosen implementation strategy [58].

- **Actors:**
  The intervention is offered by community health workers speaking the same language and belonging (or being near) to the community itself. The study’s CHWs have a similar profile to that of health workers recruited by a number of malaria control programmes, particularly in remote areas. Field activities are implemented through civil society partner organizations, who hire the CHWs and are responsible for sites’ logistics: in Suriname by the SWOS foundation, which has the purpose of developing the scientific research in health in the country; in Brazil through the NGO DPAC Fronteira, whose main activity is social mediation in health and community development at the French-Brazilian border [59].
- **Dose and temporality:**
  In the context described above, the strategy takes advantage of the regular mobility of the potential participants between the inclusion sites and gold mines, approaching the target population where and when they are easily accessible, thus overcoming the obstacles presented by the isolation of the community at their gold mines, which are often inaccessible to health teams due to security and regulatory constraints. Therefore, they will be reached on an ongoing basis rather than through one-off operations. The expected inclusion rate is between 25 and 50 participants per site per month, allowing a gradual increase of the coverage of the study’s target population. The intervention is planned to be offered for 20 months.

Additional features of the implementation strategy should be mentioned:

- **Training and supervision at the core of the implementation strategy:**
  CHWs have received comprehensive initial training allowing them to correctly carry out inclusions and follow-up [59], and benefit from continuous refresher training. The coordination team and the field supervisors ensure the fidelity of inclusions and follow-up through supervision visits and standardized evaluation, activity assessment, stocks follow-up, management of operational issues. As a part of this process, quality assurance procedures for STANDARD™ G6PD analyzer are implemented according to the PATH G6PD Operational Research Community of Practice (GORCoP) [60], and supervised inclusion processes are regularly realized including a checklist evaluating the fidelity in the G6PD testing.
- **Tailored tools elaborated through a participatory approach:**
  The content of the participants’ training as well as the information, education and communication (IEC) tools elaborated in the context of the project are the fruit of pre-intervention qualitative research about malaria knowledge and health perceptions, available at the project’s website [61]. They have been designed with the participation of the target population, to be acceptable, relevant and understandable.
- **An information system that supports the inclusion and follow-up activity:**
  The inclusion and follow-up process are supported by “smart” electronic questionnaires filled-in on tablets by the CHWs. The information system of the former Malakit project was adapted to meet the needs of CUREMA [62]. The questionnaires, based on the Open Data Kit (ODK) Collect Android application, can be used offline, and according to the information entered by CHWs, advise them on the next steps of the inclusion process, on the eligibility of participants to either services of the project, or on specific actions that need to be taken. Thanks to the weekly upload of inclusion and follow-up data to the study servers an ongoing monitoring and evaluation of data quality is performed by the coordination team.
  Moreover, a tailored smartphone application has been developed for the project. In this app, which can be used offline, participants are able to find educational videos. For participants receiving radical cure, popup notifications appear on the screen and prompt follow-up questionnaires. Data can be collected offline and sent to the study servers whenever an internet connection becomes available. Satellite based wireless connections are increasingly available even in remote gold mining sites, and regularly accessed for personal purposes by the gold miners [63].

### Evaluation

#### Design and outcomes

The CUREMA study relies on a hybrid design, assessing both population-scale effectiveness of the intervention and its implementation [57], to facilitate its translation into programme action. More precisely, this will be a type I hybrid study, testing effects of an intervention on relevant outcomes while observing and gathering information on implementation (Table 2).

**Table 2:** Objectives of the project

	Main objective	Secondary objectives
Effectiveness	To assess the evolution of malaria epidemiology before and after the intervention	To assess the evolution of the species-specific prevalence of *P*. *vivax* and* P. falciparum* among people involved in gold mining activities in the South of the Guiana Shield; To assess the evolution of the proportion of garimpeiros with a high probability of recent *P*. *vivax* infection (and probably hypnozoite carriers); To reduce the incidence of malaria cases associated with gold mining activity in the Guiana Shield, as detected by the epidemiological surveillance systems of the countries involved; To increase the proportion of *garimpeiros* who adequately take anti-malarial treatment when they fall ill in illegal mining sites in French Guiana
Implementation	To assess the actual reach (penetration) of the intervention overall and for each intervention service	To assess the acceptability of the intervention for target public and field workers To assess the relevance of the intervention for the intervention’s stakeholders (target population, field workers, researchers, policy-makers) To assess the feasibility of the intervention for the field workers and researchers involved in the project To assess the adherence to the primaquine posology among asymptomatic individuals; To assess the safety of medicines used on a community scale; To evaluate the effectiveness of the health education activity carried out during the intervention; To assess the acceptability and feasibility of digital tools (smartphone app); To evaluate the quality and effectiveness of the training received by facilitators; To assess the fidelity of the inclusion and follow-up process; To assess the cost of the intervention; To assess the health situation of garimpeiros and additional health needs beyond malaria elimination; To assess facilitating factors as well as barriers to delivering such an intervention in a pre-elimination setting and community involvement to be taken into account for further implementation

The effectiveness of the intervention on malaria transmission is evaluated by a pre/post quasi-experimental design. Therefore, the main outcome of the study is the variation of the proportion of people carrying *Plasmodium* spp. parasites by ultrasensitive PCR measured before and after the intervention. To support the interpretation of this outcome, the evolution of malaria epidemiology in the region over the study period will be assessed by: (1) data from the surveillance systems of the three countries involved in the project; (2) the analysis of the evolution of serologically positivity rate for *P. vivax*; (3) collection of dry blood spot (DBS) samples for each participant in the intervention (usPCr and Pv serology). This will allow a modelling of malaria epidemiological fluctuations occurring in the region during the intervention.

The main outcome chosen to evaluate the implementation of the intervention is its penetration [64] within the target population, i.e. the proportion of the target population included in the intervention at the end of the study period. The effectiveness of the intervention is in fact closely dependent on its actual execution and on the coverage of the target population. To put this outcome into context and to provide food for thought about possible scale-up or transferability, acceptability, safety, appropriateness, feasibility, fidelity and sustainability will be assessed by quantitative data and qualitative surveys [64].

**Fig. 4:**
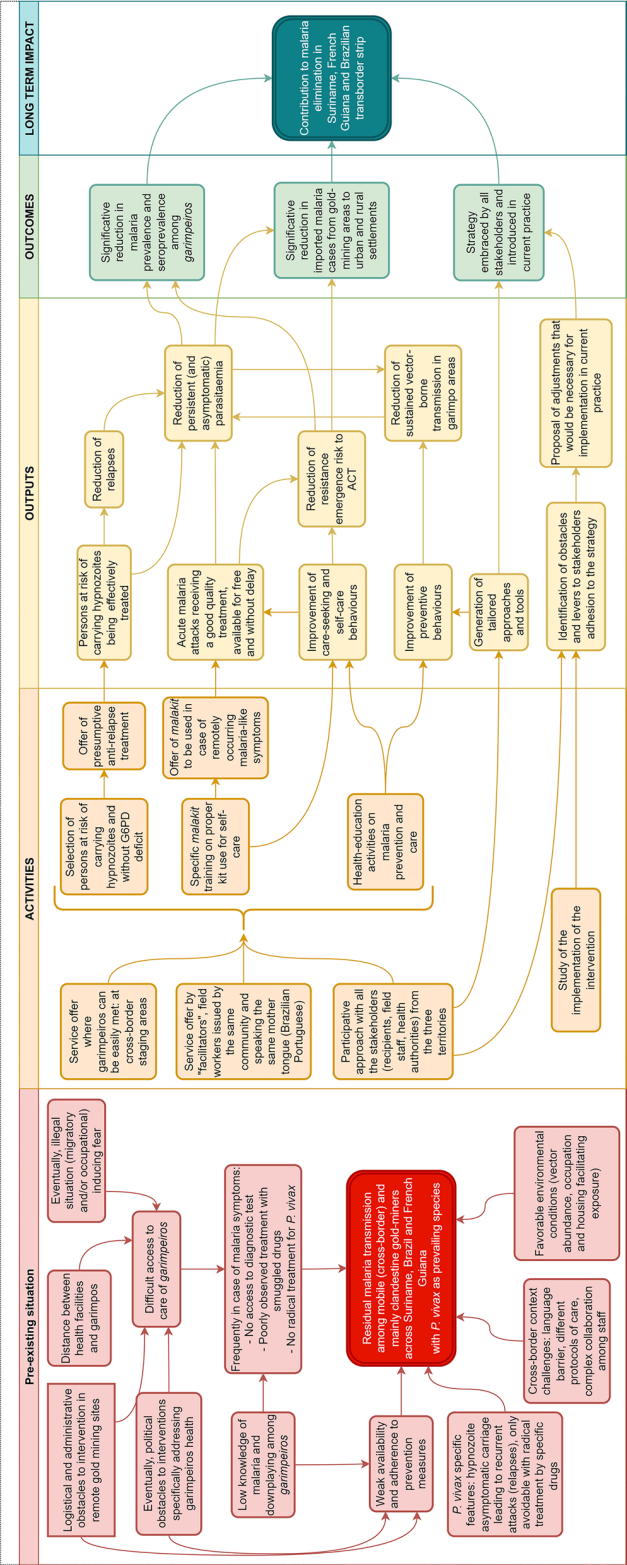
Intervention's theory

The underlying assumptions about how these objectives should be achieved are set out in the logic model proposed in Fig. 4.

#### Data sources

**Fig. 5:**
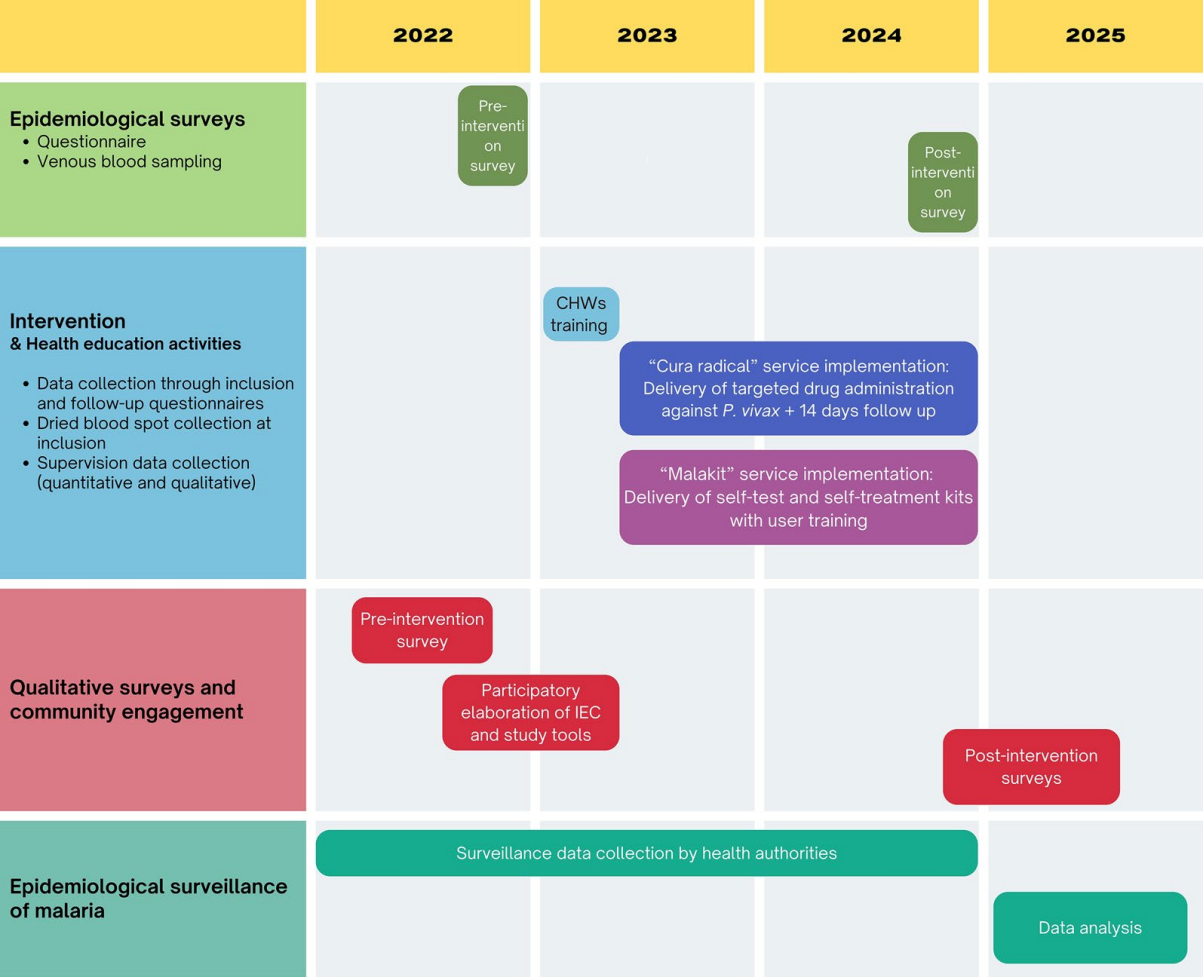
CUREMA project design and timeline

Data for evaluation are provided through different study components which articulation is illustrated in Fig. 5.

- **The intervention:**
  Inclusion and follow-up questionnaires provide information on the number of participants, their socio-demographic and occupational profile, the actual delivery of the services (dependent on participants’ choice and eligibility), the adherence to the treatments and their safety. DBS are collected for all the participants undergoing fingerpick for G6PD test or self-test training. Data generated from participants follow-up and from AE investigations will allow to produce evidence on the safety of the intervention. For the intervention, no sample size has been defined from a statistical perspective: its target is to include as many persons as feasible. Between 2500 and 5000 participants are expected during the study’s period, in the same order of magnitude as the Malakit project [39]. A non-inclusion registry collects anonymous information about individuals that did not wish to participate in the study or did not meet the inclusion criteria, contributing to the acceptability evaluation. Important additional data for the evaluation of the implementation are the information produced by the supervision activities (check-lists, audits) and the evaluation of the training of facilitators performed during the initial session [59].
- **The pre-and post-intervention epidemiological surveys:**
  Two cross-sectional surveys take place at inclusion sites before and at the end of the intervention implementation, during the same period of the year (during the last quarters of 2022 and 2024) in order to limit potential biases associated with seasonality. The participants are selected among individuals having left an illegal gold mine located in FG within the past seven days [20, 39]. These surveys include a detailed questionnaire about recent malaria history and mobility, a clinical examination and a venous blood sample. The proportion of malaria parasite carriage will be assessed by a *Plasmodium* us-qPCR (ultra-sensitive quantitative polymerase chain reaction) [65], with species-specific probes for *P. vivax*, *P. falciparum* and *Plasmodium malariae* asexual and sexual forms. *Plasmodium vivax* serology by Luminex assay will be performed following methodology described by Longley et al. [66], in order to assess recent (and thus potentially latent) infections, as well as medium-term transmission trends [66–69]. The biological collection will be stored at biobank Centre de Ressources Biologiques Amazonie in Cayenne.
  The sample size requirements have been calculated based on the univariate analysis of the main effectiveness outcome: with a hypothetic pre-intervention all-species prevalence of 2%, and a target 75% reduction after two years of intervention (leading to a post-intervention prevalence of 0.5%), 860 participants should be included in each survey in order to perform this comparison with a two-sided 0.05 alpha risk and a 0.8 beta risk.
- ***Qualitative research :***
  Qualitative surveys are performed before, during and after the intervention by a social science researcher. The aim of these surveys is to support community engagement and to analyse the specific constraints and levers of the intervention under study and the pre-elimination context, to understand which elements could influence the success or the failure of the intervention and implementation strategy under evaluation. The qualitative research addresses three groups involved in the study: (i) the target population of the intervention, (ii) the field workers who participate in the inclusion and supervision activities, (iii) scientific and institutional (technical officers and decision-makers) stakeholders of the study.
  Interviews and discussion groups are proposed at different times to all stakeholders of the study. Participant and non-participant observation are conducted to collect descriptive data on context, behaviours, interactions and dynamics, experiences, and will allow researchers to better describe and interpret the data.
- **National and regional epidemiological surveillance data:**
  Data from the malaria programmes surveillance systems of French Guiana, Brazil and Suriname regarding cases notified, according to their origin and (while available) occupational category will allow to evaluate the general context of the regional malaria epidemiology.

## Results

The *Centre d’Investigation Clinique Antilles-Guyane* is an INSERM (Institut National de la Santé et de la Recherche Médicale) research unit based at Cayenne Hospital and is the sponsor of the CUREMA study. Key scientific partners include the SWOS and the Fundação Oswaldo Cruz (FIOCRUZ): these institutions host the principal investigators responsible for inclusions in Suriname and Brazil, respectively. Major scientific collaborations with the Pasteur Network support the project, providing expertise in the molecular biology and immunology of malaria. In Brazil, collaboration with the NGO DPAC-Fronteira brings to the project significant experience in social mediation, health education and mobilization, and empowerment of vulnerable communities.

The project is supported by the health authorities competent for the three territories and their respective malaria elimination programmes. These institutions are also part of the steering and scientific committee of the project. Besides the financial or in-kind support from health authorities, the CUREMA project is also funded by the European Funds of Regional Development from PCIA (Programme de Coopération Interreg Amazonie, SYNERGIE 7128 and 8754).

The project received ethical clearance from the Ministry of Health of Suriname (CMWO 005/22), the Fiocruz ethics committee (CEP 5.210.165) and the National ethical committee for health research of Brazil (CONEP 5.507.241). It also complies with the European Regulation on Data Protection. A Data and Safety Monitoring Board (DSMB) has been established to provide external monitoring of the study, specifically advising the investigators and the sponsor about potential safety concerns. Field implementation of the study started in the last quarter of 2022 and is planned to take 27 months (Fig. 5). Results will be available at the end of 2025.

## Discussion

The CUREMA project aims at evaluating a complex intervention [70, 71]: several components make up the CUREMA intervention per se, including pharmacological intervention and health education activities; the context of the intervention is complex, being cross-border, characterized by challenging operational and logistical aspects due to the Amazonian environment and the fragility of infrastructures, by the interaction of numerous actors belonging to a multicultural and multisector context; the implementation of the intervention and its effectiveness can be significantly influenced by the epidemiological, migratory, political, economic and climatic context of the region. In this context, the authors' objectives cannot be limited to a simple evaluation of effectiveness, but it is fundamental to address the question of for whom, when, why and how this intervention can be effective and relevant [70].

In terms of intervention’s environment, the CUREMA project is the natural continuation of the Malakit project, which was carried out between 2018 and 2020 by the same nexus of scientific, operational and institutional partners, and whose experience CUREMA capitalizes on. Both projects were born from a virtuous dynamic in which these players, belonging to different professional backgrounds and the three countries in the Region, sought to collectively build creative solutions to common challenges. The reduction in the level of malaria transmission in the three territories is an important contextual element. On the one hand, the participation of Suriname and French Guiana in the E-2025 initiative, as well as the Brazilian government’s commitment to eliminating malaria transmission in the Amazon by 2035, are levers of increased political support to implement ambitious interventions to achieve these goals. On the other hand, paradoxically, the decreasing number of malaria cases may lead to a gradual demotivation of the community and of field professionals involved, whose reduced level of commitment may jeopardize the ability of these interventions to produce the expected effects. The evaluation of the CUREMA project will need to take these contextual factors into account, in order to understand the factors at play in the implementation of the project and its acceptance.

The CUREMA project and the previous MALAKIT project have been conceived with two core principles in mind. The first is medical neutrality and impartiality, i.e. the fact that access to health should be universal, a key principle of medical ethics and humanitarian law [72, 73]. The study population has de facto restricted access to healthcare due to its clandestine status, and the aim of the project’s approach is to restore this access, at least in the fight against malaria. Secondly, the “harm reduction” perspective, which involves a non-judgemental approach to promoting health and reducing the harmful effects of a not easily avoidable exposure [74]. In this case, the exposure is represented by the activity of gold mining, which has very complex social and economic determinants, and on which the health sector is unable to intervene. Precautions must be taken when working in this context. As mentioned above, project’s actions are carried out in neutral contexts, where participants are not in conditions of illegality at the time of inclusion. It is also important to note that these places are widely known in literature and by the authorities [36], and not revealed by the project. Additionally, data collection carefully avoids gathering information on possible criminal activities (including human trafficking, violence, drug trafficking). Finally, it is important to emphasize that the majority of the study population, although in irregular migratory conditions and involved in unauthorized mineral exploitation, are simple “workers” who are not involved in criminal activities [36]. They also suffer from very vulnerable socio-economic and health conditions [38, 75].

The project team has gradually built up a relationship of trust with the ASGM communities [76]. Participation in the study by this “hidden” population is facilitated by the fact that the intervention is carried out in “neutral” locations, through work with members of the community, and by the history of collaboration and trust established by the research team and field partners over more than a decade. This has helped to nurture a community-engagement approach with a dual objective: to produce services and results considered relevant by the target community, and to strengthen its involvement and awareness around malaria elimination efforts. The CUREMA project intervention and evaluation components have been designed by researchers and malaria experts, but the opinion of the target population was sought throughout the development process, and most project tools (questionnaires, application, educational material) were developed with their direct participation. In this regard, the participation of the community is situated, on the continuum described by Sanderson and colleagues, between consultation and cooperation [77]. Nevertheless, the fact that the project proposal is not generated per se by a community approach, could have the effect that the community does not feel it is relevant and does not embrace it, despite efforts to improve their participation.

Interventional health studies can be qualified according to their characteristics on a continuum between the attributes explanatory and pragmatic [78]. In explanatory trials the object of evaluation is the drug (or technology) per se, which is compared to placebo or standard of care under ‘‘optimal’’ and balanced conditions, in order to identify its specific role in the evolution of a health state. In pragmatic trials, the intervention is evaluated under conditions as close to real life as possible, in order to generate information about its effective applicability [78]. The drugs used in the study have already been the subject of explanatory studies, or even are already included in national recommendations for the treatment of acute malaria episodes. The CUREMA project proposes an approach that changes the indications or modalities of such treatments, as well as the mode of recruitment of patients receiving the drugs. However, it did not seem relevant to carry out an explanatory study for this approach, and on the contrary, a pragmatic design seems more interesting: due to the nature of the intervention, its applicability in real conditions is indispensable, and a result obtained in controlled ‘‘optimal’’ conditions would not have any added value in terms of decision support for the health authorities. For example, a study recruiting patients in a health facility by medical or paramedical professionals (rarely available in the target locations), with an ‘‘ideal’’ in person clinical and biological follow-up (which does not take into account the high mobility of the target population), would not only make this intervention difficult to transpose to the reality of places with residual transmission in endemic countries, but would also end up missing the very target of the intervention. Field health workers with a similar profile are in charge of treating malaria and other communicable diseases on a daily basis in many countries of the world (including Brazil and Suriname) [79–83]. In Suriname, a partnership with the National Malaria Programme allow to hire as collaborators of the project the same CHWs involved in the programme’s activities. However, setting up an intervention in a research project is by its very nature different from scaling it up in a healthcare system, in terms of organization, administrative, political and logistical constraints, and funding capacity [84]. These limitations will need to be taken into account when assessing the transferability of the intervention.

The effectiveness evaluation the authors are interested in measuring is the impact of the intervention on malaria transmission at a population level. Field conditions (limited number of inclusion sites, high mobility of the target population across the region between different gold mining areas) would not allow the implementation of a cluster randomized trial, as well as of other types of controlled designs. Effectiveness evaluation with a non-randomized design without a control group will require caution when interpreting the main outcome results. Potential confounding factors must be considered in the interpretation of the results, as changes in malaria prevalence could be linked to external factors such as changes in mobility patterns, environmental variations, or evolutions in malaria control programme activities. Contextual data on the epidemiological, health, environmental, economic and political context will provide additional insight.

Evaluation of implementation (using quantitative and qualitative data) will also allow to interpret the results of the impact assessment more adequately: to what extent was the intervention implemented satisfactorily, and what are the factors favouring or hindering its implementation in general and on specific aspects. The triangulation of these elements will help to understand whether, how and why the intervention worked in the study’s context. This will also provide a basis for imagining whether and how it might work in similar contexts.

Another very important point that will be analysed with CUREMA is the risk–benefit balance (real and perceived) of this targeted drug administration against *P. vivax* silent carriage. On the one hand, mass drug administration (MDA) aiming at eliminating *P. vivax* has already been carried out in the past [85, 86]. However, these actions were fraught with a dubious risk–benefit balance because of the high proportion of people unnecessarily treated, especially in contexts of medium–low endemicity. An alternative to MDA would be to carry out targeted treatment of people who are seropositive for *P. vivax* (serological test-and-treat, seroTaT), serology being used as a proxy for recent infection with *P. vivax* and of the carriage of hypnozoites. This has been used in the past for malaria elimination in southern Brazil, and has been advocated as a mass strategy more recently in several modelling papers [66, 87]. However, the unavailability of rapid serological tests that can be used in the field implies that this strategy is not currently applicable to such an isolated and highly mobile population. The proposal for a targeted drug administration strategy based on epidemiological criteria (recent individual history compatible with asymptomatic carriage) is an innovative compromise aimed at improving the risk–benefit balance using simple methods available everywhere and with low cost. The epidemiological criteria used in the study will be compared with serology retrospectively to assess their performance.

The risk of error in the delivery of radical cure intervention by community health workers will be assessed, particularly the risk of delivering 8-aminoquinoline to persons with contra-indications such as G6PD deficiency. To prevent this risk, a comprehensive initial training program [59] has been set up, as well as periodic supervision and refresher trainings to guarantee the quality of the test performance. The choice of whether to deliver the radical treatment is accompanied by the electronic inclusion form, which provides recommendations in relation to the level of G6PD and other contraindications explored. Although project’s field workers are not professionals with specific training in laboratory techniques, other experiences in Brazil shows that field performance of this test by community health workers without formal qualifications is possible and safe [54, 55]. An unpublished study was also carried out in Suriname on the feasibility of testing by community health workers in the malaria programme (the same ones recruited for CUREMA), with satisfactory results (personal communication with Dr. S. Vreden). The frequency and severity of adverse events recorded during the participants’ follow-up will assess the risk incurred by participants. The balance as perceived by participants will be evaluated during qualitative surveys carried out after the intervention. All these elements will thus contribute to the analysis of the risk–benefit balance of this service offered under field conditions, in low to moderate malaria transmission settings to asymptomatic persons.

## Conclusions

The CUREMA study will provide an evaluation of a new intervention for hard-to-reach populations, who represent the main challenge for countries approaching the elimination of malaria. These results will, therefore, be disseminated and used to inspire solutions in similar realities, for example in Latin America and Asia [13, 16, 24, 28, 37], in the context of transfer to health systems or of further scientific evaluation (including consolidation of the effectiveness results, or medico-economic assessment). Furthermore, the same intervention could also be considered for the management of epidemic phenomena when logistical, political and/or administrative constraints make it impossible to set up on-site interventions (including clandestine populations, war contexts). These results would, therefore, prove extremely valuable to face the challenges of malaria elimination in a growing number of countries [88].

## Data Availability

Not applicable.
